# Okanin, effective constituent of the flower tea *Coreopsis tinctoria*, attenuates LPS-induced microglial activation through inhibition of the TLR4/NF-κB signaling pathways

**DOI:** 10.1038/srep45705

**Published:** 2017-04-03

**Authors:** Yue Hou, Guoxun Li, Jian Wang, Yingni Pan, Kun Jiao, Juan Du, Ru Chen, Bing Wang, Ning Li

**Affiliations:** 1 College of Life and Health Sciences, Northeastern University, Shenyang 110819, China; 2 School of Pharmaceutical Engineering, Shenyang Pharmaceutical University, Shenyang 110016, China; 3 School of Traditional Chinese Materia Medica, Key Laboratory of Structure-Based Drug Design and Discovery, Shenyang Pharmaceutical University, Ministry of Education, Shenyang 110016, China

## Abstract

The EtOAc extract of *Coreopsis tinctoria* Nutt. significantly inhibited LPS-induced nitric oxide (NO) production, as judged by the Griess reaction, and attenuated the LPS-induced elevation in iNOS, COX-2, IL-1β, IL-6 and TNF-α mRNA levels, as determined by quantitative real-time PCR, when incubated with BV-2 microglial cells. Immunohistochemical results showed that the EtOAc extract significantly decreased the number of Iba-1-positive cells in the hippocampal region of LPS-treated mouse brains. The major effective constituent of the EtOAc extract, okanin, was further investigated. Okanin significantly suppressed LPS-induced iNOS expression and also inhibited IL-6 and TNF-α production and mRNA expression in LPS-stimulated BV-2 cells. Western blot analysis indicated that okanin suppressed LPS-induced activation of the NF-κB signaling pathway by inhibiting the phosphorylation of IκBα and decreasing the level of nuclear NF-κB p65 after LPS treatment. Immunofluorescence staining results showed that okanin inhibited the translocation of the NF-κB p65 subunit from the cytosol to the nucleus. Moreover, okanin significantly inhibited LPS-induced TLR4 expression in BV-2 cells. In summary, okanin attenuates LPS-induced activation of microglia. This effect may be associated with its capacity to inhibit the TLR4/NF-κB signaling pathways. These results suggest that okanin may have potential as a nutritional preventive strategy for neurodegenerative disorders.

*Coreopsis tinctoria* Nutt. is native to North America and has now spread worldwide. In China, it originally grows on the Kunlun Mountain at an altitude above 3000 m in southern Xinjiang. Because of its considerable scientific and commercial use, it is now cultivated widely in Shandong, Hebei and Henan provinces. Known locally as “snow chrysanthemum” or “snow tea”, it is traditionally used as a tea-like beverage and is a popular health supplement to prevent cardiovascular disease^1^,^2^,^3^,^4^. In our previous research, its significant anti-neuroinflammatory effect attracted our attention^5^.

In the brain, neuroinflammatory responses are important in protecting against infections and neuronal damage^6^,^7^, but these responses can cause pathological neuronal changes if they are not properly controlled^6^,^7^,^8^. Microglial cells, which are the macrophages in the brain, play a major role in the production of pro-inflammatory mediators in response to inflammatory stimuli. However, over-activation of microglia can lead to over-production of mediators, including tumor necrosis factor α (TNF-α), interleukin 6 (IL-6), interleukin-1β (IL-1β) and nitric oxide (NO), which are thought to cause neuronal injuries similar to those observed in neurodegenerative disorders^9^,^10^,^11^. Although there is growing evidence for a role of over-activated microglia in neurodegenerative disorders, there are still no effective treatments for or preventions against neurodegeneration.

We are interested in identifying naturally occurring plant compounds that have anti-neuroinflammatory properties^6^. We selected the flower tea *Coreopsis tinctoria* Nutt. as a possible source of compounds. Here, we report the anti-neuroinflammatory effect of okanin, a major effective constituent of *Coreopsis tinctoria* Nutt, and describe the possible mechanisms that may underlie its effects.

## Results

### Effects of extracts of *C. tinctoria* Nutt on LPS-induced NO production and iNOS expression

**Figure 1:**
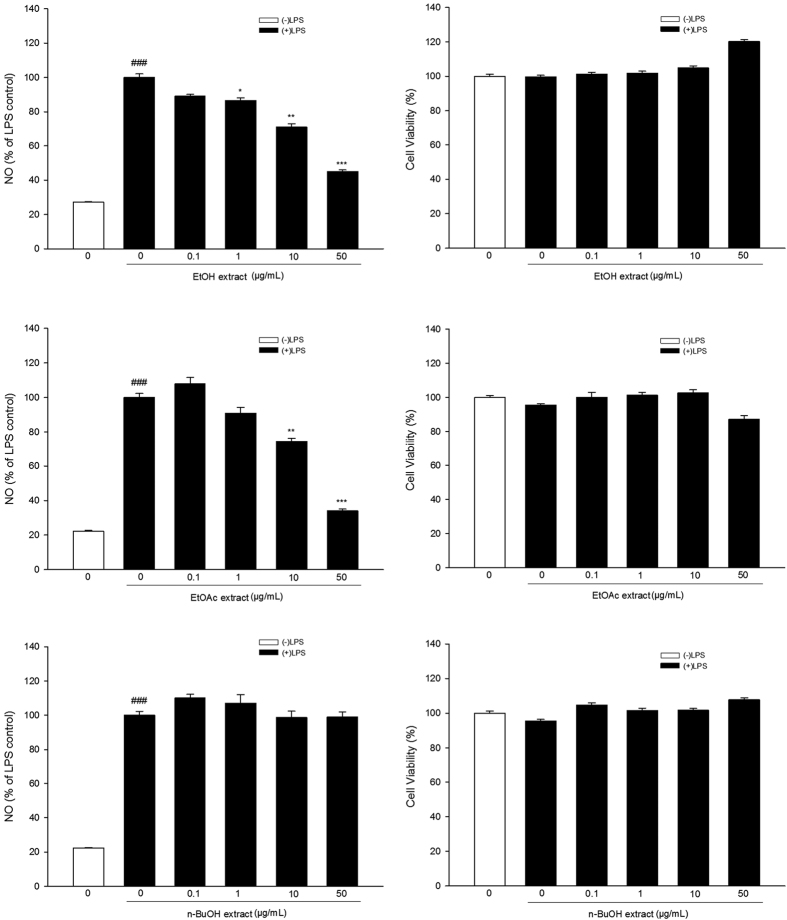
Anti-inflammatory activity of *Coreopsis tinctoria* Nutt. in BV-2 microglial cells. BV-2 microglial cells were treated with extracts of *Coreopsis tinctoria* Nutt. in the presence of LPS (100 ng/mL) for 24 h at the indicated concentrations. Aliquots of the culture supernatants were removed and analyzed for nitrite production. Cell viability was determined by MTT assay. Data are expressed as means ± SEM (n = 3). ^###^P < 0.001 compared with the untreated control group, *P < 0.05, **P < 0.01 and ***P < 0.001 compared with the cells treated with LPS only. (EtOH: ethanol, EtOAc: ethyl acetate, n-BuOH: *n*-butanol, NO: nitric oxide, LPS: lipopolysaccharide).

The total EtOH extract (1, 10, 50 μg/ml) of *Coreopsis tinctoria* Nutt significantly inhibited LPS-induced NO production (control group: (27.1 ± 0.3)%, LPS group: (100.0 ± 2.0)%, EtOH extract 0.1 μg/ml group: (89.1 ± 1.0)%, EtOH extract 1 μg/ml group: (86.4 ± 1.6)%, EtOH extract 10 μg/ml group: (71.0 ± 1.9)%, EtOH extract 50 μg/ml group: (45.0 ± 0.9)%), Fig. 1). Further study revealed that the EtOAc extracts (10, 50 μg/ml) significantly inhibited LPS-induced NO production (control group: (22.2 ± 0.5)%, LPS group: (100.0 ± 2.3)%, EtOAc extract 0.1 μg/ml group: (107.8 ± 3.7)%, EtOAc extract 1 μg/ml group: (90.9 ± 3.3)%, EtOAc extract 10 μg/ml group: (74.4 ± 1.8)%, EtOAc extract 50 μg/ml group: (34.0 ± 1.0)%), Fig. 1), while the n-BuOH extract did not (control group: (22.2 ± 0.5)%, LPS group: (100.0 ± 2.3)%, n-BuOH extract 0.1 μg/ml group: (110.0 ± 2.1)%, n-BuOH extract 1 μg/ml group: (107.1 ± 5.1)%, n-BuOH extract 10 μg/ml group: (98.7 ± 3.8)%, n-BuOH extract 50 μg/ml group: (98.9 ± 2.9)%), Fig. 1). We also tested whether the extracts were cytotoxic to BV-2 cells. MTT assay results showed that none of the extracts were cytotoxic.

**Figure 2:**
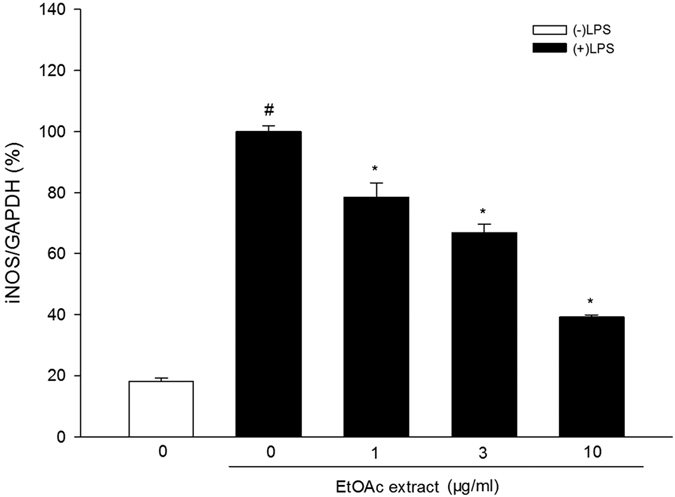
Effect of EtOAc extract of *Coreopsis tinctoria* Nutt. on LPS-induced iNOS mRNA expression in BV-2 microglial cells. Cells were pretreated with the extract (1, 3, 10 μg/ml) for 2 h and then stimulated with LPS (100 ng/mL) for 4 h. The iNOS mRNA levels were measured by qRT-PCR. Data are expressed as means ± SEM (n = 4). ^#^P < 0.05 compared with the control group (untreated cells), *P < 0.05 compared with the cells treated with LPS only.

The effects of the EtOAc extracts on LPS-induced iNOS mRNA expression in BV-2 microglial cells were investigated by quantitative real-time PCR (qRT–PCR). Shapiro-Wilk test showed that the data were not normally distributed. Kruskal-Wallis test showed significant difference among different groups [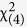 = 17.899, P < 0.01, Fig. 2]. Mann-Whitney test further showed that the cells treated with LPS alone showed greatly increased iNOS mRNA expression compared to the control group (control group: (18.2 ± 1.1)%, LPS group: (100.0 ± 1.9)%), while the EtOAc extract significantly inhibited LPS-induced iNOS mRNA expression (1 μg/ml group: (78.6 ± 4.7)%, 3 μg/ml group: (67.0 ± 2.7)%, 10 μg/ml group: (39.2 ± 0.7)%).

### Effect of the EtOAc extract of *C. tinctoria* Nutt on LPS-induced mRNA expression of inflammatory mediators

**Figure 3:**
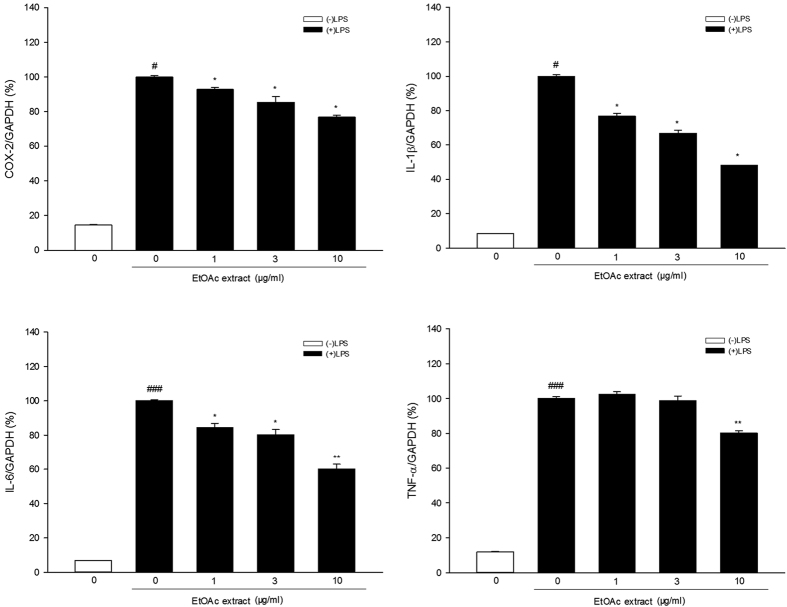
Effect of EtOAc extract of *Coreopsis tinctoria* Nutt. on LPS-induced mRNA expression of inflammatory mediators in BV-2 microglial cells. Cells were pretreated with the extract (1, 3, 10 μg/ml) for 2 h and then stimulated with LPS (100 ng/mL) for 4 h. The mRNA levels were measured by qRT-PCR. Data are expressed as means ± SEM (n = 4). ^#^P < 0.05, ^###^P < 0.001 compared with the untreated cells (control group), *P < 0.05, **P < 0.01 compared with the cells treated with LPS alone (LPS group).

In the experiment to test COX-2 mRNA expression, Shapiro-Wilk test showed that the data were not normally distributed. Kruskal-Wallis test showed significant difference among different groups [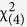 = 17.137, P < 0.01, Fig. 3]. Mann-Whitney test further revealed that COX-2 mRNA expression was much higher in the LPS-only group than in the control group (control group: (14.5 ± 0.4)%, LPS group: (100.0 ± 0.8)%), while LPS-induced COX-2 mRNA expression was significantly inhibited by the EtOAc extract (1 μg/ml group: (93.0 ± 1.2)%, 3 μg/ml group: (85.5 ± 3.4)%, 10 μg/ml group: (76.9 ± 1.1)%) at all three concentrations tested.

In the experiment to test IL-1β mRNA expression, Shapiro-Wilk test showed that the data were not normally distributed. Kruskal-Wallis test showed significant difference among different groups [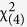 = 18. 327, P < 0.01, Fig. 3]. Mann-Whitney test showed that IL-1β mRNA expression was much higher in BV-2 cells treated with LPS alone compared to the control group, while the EtOAc extract significantly inhibited LPS-induced IL-1β mRNA expression at all concentrations tested (control group: (8.5 ± 0.1)%, LPS group: (100.0 ± 0.8)%, 1 μg/ml group: (76.9 ± 1.7)%, 3 μg/ml group: (66.7 ± 1.9)%, 10 μg/ml group: (48.0 ± 0.1)%).

One-way ANOVA analysis revealed significant differences between the groups in the experiment to test IL-6 mRNA expression [F(4, 15) = 291.884, P < 0.001, Fig. 3]. Dunnett’s T3 test showed that IL-6 mRNA expression was much higher in the BV-2 cells treated with LPS alone than in the control cells, and the EtOAc extract significantly inhibited LPS-induced IL-6 mRNA expression at all concentrations tested (control group: (6.8 ± 0.1)%, LPS group: (100.0 ± 0.6)%, 1 μg/ml group: (84.5 ± 2.4)%, 3 μg/ml group: (80.3 ± 3.0)%, 10 μg/ml group: (60.1 ± 2.8)%).

Regarding TNF-α mRNA expression, one-way ANOVA analysis revealed significant differences between the groups [F(4, 15) = 671.675, P < 0.001, Fig. 3]. Dunnett’s T3 test showed that TNF-α mRNA expression was greatly increased in the BV-2 cells treated with LPS alone compared to the untreated control cells, and the EtOAc extract (10 μg/ml) significantly inhibited LPS-induced TNF-α mRNA expression (control group: (11.9 ± 0.3)%, LPS group: (100.0 ± 1.0)%, 1 μg/ml group: (102.5 ± 1.5)%, 3 μg/ml group: (98.8 ± 2.8)%, 10 μg/ml group: (80.3 ± 1.2)%).

### Effects of the EtOAc extract of *C. tinctoria* Nutt on LPS-induced microglia activation *in vivo*

We next determined the anti-neuroinflammatory effect of the EtOAc extract *in vivo*. Mice were pre-treated orally with the extract, then microglial activation was induced by LPS administered intracerebroventricularly. The microglial response was detected immunohistochemically using an antibody against Iba-1, a protein that is strongly up-regulated in activated microglia.

**Figure 4:**
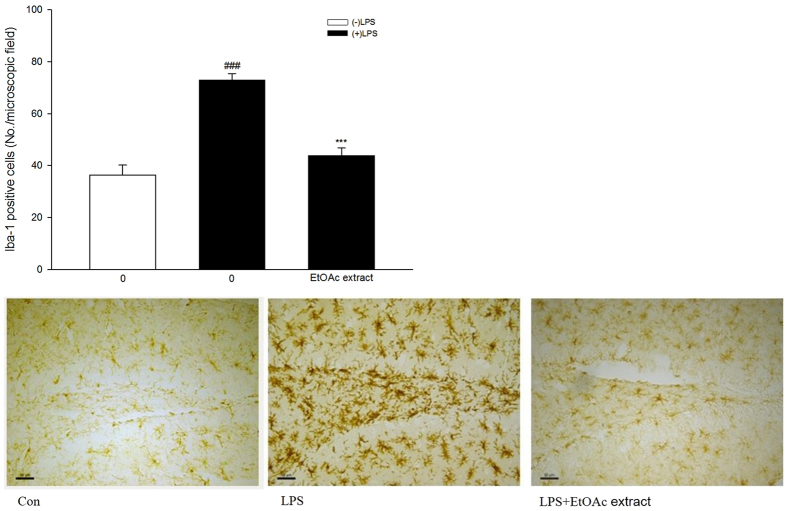
Effect of EtOAc extract of *Coreopsis tinctoria* Nutt. (200 mg/kg, i.g.) on Iba-1 immunohistochemical staining of the mouse hippocampal region on the 3^rd^ day after intracerebroventricular injection of LPS. Representative images are provided and the scale bars in the images represent 50 μm. Data are expressed as mean ± SEM (n = 14). ^###^P < 0.001 compared with the control group; ***P < 0.001 compared with the LPS group.

Shapiro-Wilk test showed that the data were not normally distributed. Kruskal-Wallis test showed significant difference among different groups [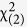 = 26.825, P < 0.001, Fig. 4]. Mann-Whitney test further indicated that mice treated with LPS had significantly more Iba-1-positive cells compared to the control group (P < 0.001). Mice treated with the EtOAc extract (200 mg/kg) and LPS had significantly fewer Iba-1 positive cells than mice treated with LPS alone (control group: 36.4 ± 3.8, LPS group: 73.0 ± 2.5, EtOAc extract group: 43.8 ± 2.9).

### The content of okanin in EtOH, EtOAc and n-BuOH extracts of *C. tinctoria* Nutt

**Figure 5:**
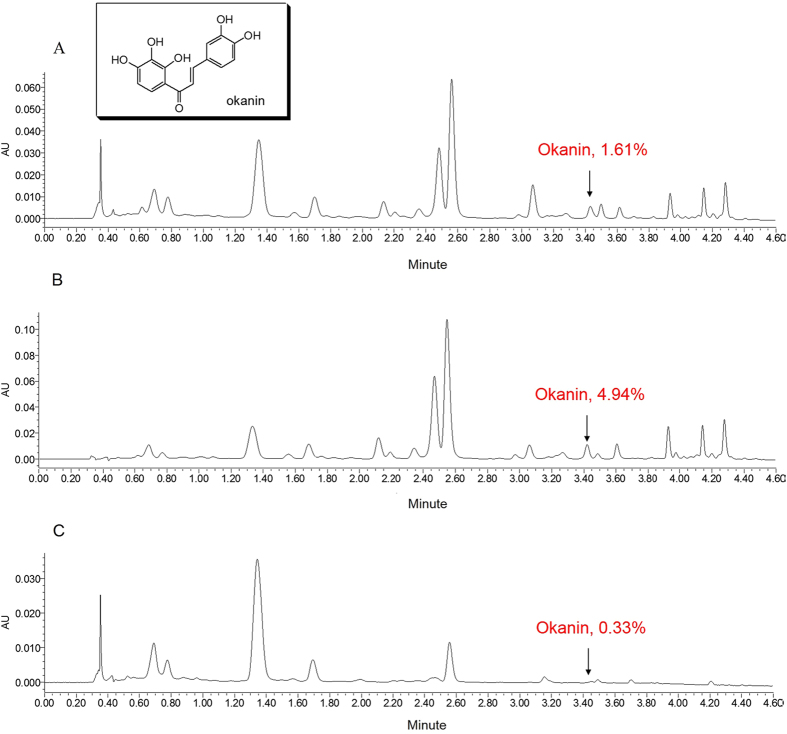
(**A**) UPLC chromatogram of EtOH extract of *C. tinctoria* at 280 nm. (**B**) UPLC chromatogram of EtOAc extract of *C. tinctoria* at 280 nm. (**C**) UPLC chromatogram of n-BuOH extract of *C. tinctoria* at 280 nm.

The okanin used in our experiment was prepared from the EtOAc extract of the flower tea *C. tinctoria* Nutt. by means of a chromatography method. Its purity was identified as 99.1% by HPLC. The content of okanin in EtOH, EtOAc and *n*-BuOH extracts was determined as 1.61%, 4.94% and 0.33%, respectively (Fig. 5A,B and C) by means of UPLC chromatography (Waters Acquity UPLC H-Class system equipped with a Sample Manager-FTN, Quaternary Solvent Manager, PDA Detector and Empower software). Therefore, okanin was concentrated most effectively in the EtOAc extract.

### Effects of okanin on LPS-induced iNOS expression

**Figure 6:**
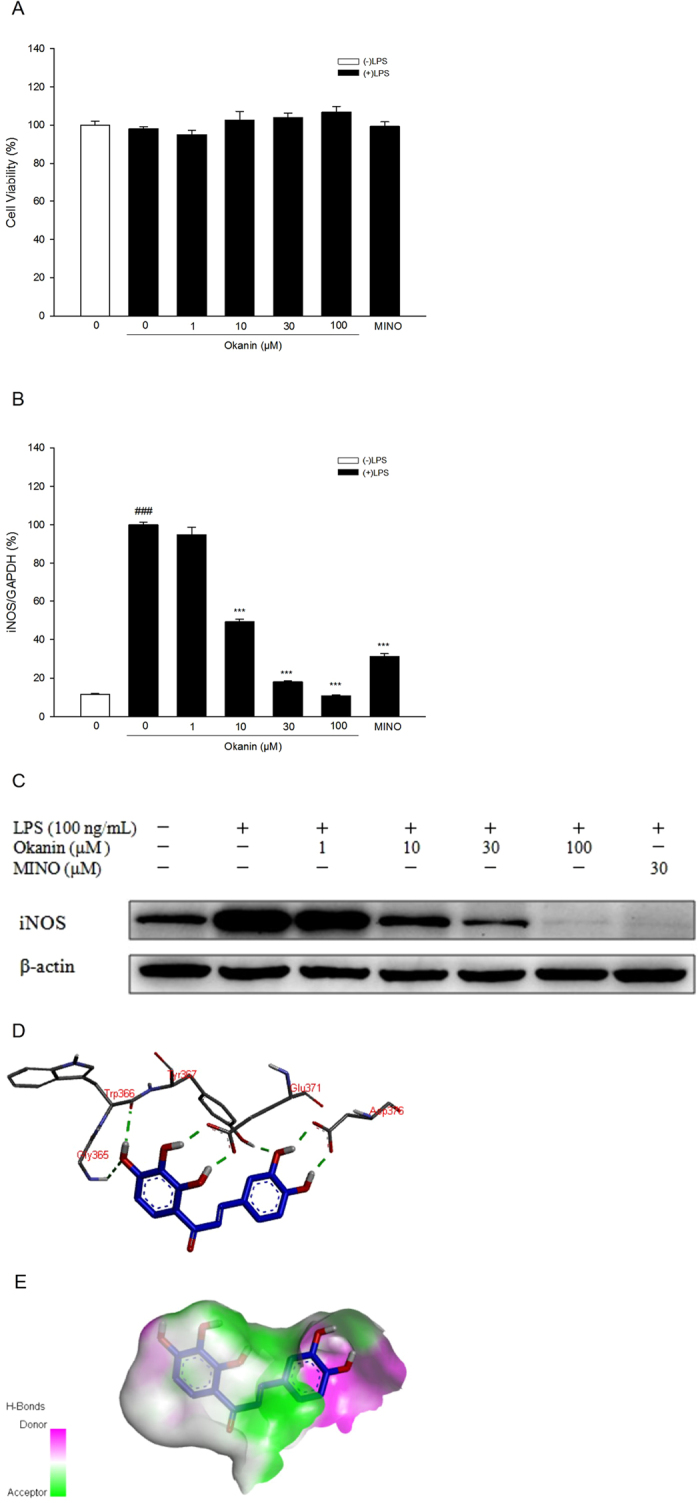
(**A**) Effect of okanin on cell viability. (**B**) Effect of okanin on LPS-induced iNOS mRNA expression in BV-2 microglial cells (n = 4). (**C**) Effect of okanin on LPS-induced iNOS protein expression in BV-2 microglial cells. Full-length blots are presented in Supplementary Figure 1. (**D**) H-bonds between okanin and iNOS pocket. (**E**) Hydrophobicity between okanin and pockets atoms. ^###^P < 0.001 compared with the control group; ***P < 0.001 compared with the LPS group.

At the beginning, MTT assay was carried out. The results showed that okanin did not reduce the cell viability (Fig. 6A), excluding a possible effect of reduced viability on mRNA or protein expression in the following experiments.

In terms of iNOS mRNA expression, one-way ANOVA analysis revealed significant differences between the groups [F(6, 21) = 478.932, P < 0.001, Fig. 6B]. Dunnett’s T3 test showed that iNOS mRNA levels were much higher in the cells treated with LPS alone than in the control group, while okanin (10, 30,100 μM) significantly inhibited iNOS mRNA expression induced LPS (control group: (11.6 ± 0.4)%, LPS group: (100.0 ± 1.3)%, 1 μM group: (94.8 ± 3.9)%, 10 μM group: (49.3 ± 1.3)%, 30 μM group: (18.0 ± 0.7)%, 100 μM group: (10.6 ± 0.6)%, minocycline group: (31.2 ± 1.5)%). As shown by Western blot analysis, okanin markedly attenuated LPS-induced iNOS protein level (Fig. 6C).

A molecular docking study was performed to clarify the mode of action of okanin on iNOS. As shown in Fig. 6D and E, okanin formed several hydrogen bonds with Gly365, Trp366, Tyr367, Glu371 and Asp376 of iNOS, and fitted well within the iNOS binding pocket.

### Effects of okanin on LPS-induced production and mRNA expression of inflammatory mediators

**Figure 7:**
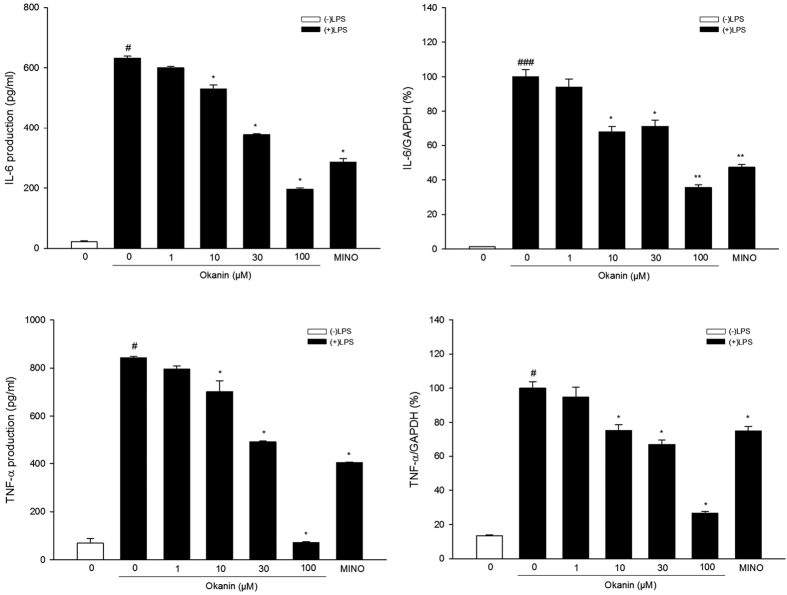
Effects of okanin on LPS-induced IL-6 and TNF-α production and mRNA expression in microglial cells. BV-2 microglial cells were pretreated with okanin (1, 10, 30, 100 μM) for 2 h and then stimulated with LPS (100 ng/mL) for 24 h, and the production of IL-6 and TNF-α in cell supernatants were measured using ELISA kits. Total RNA was isolated 4 h after LPS treatment and the mRNA levels of IL-6 and TNF-α were measured by qRT-PCR. Data are expressed as means ± SEM (n = 4). ^#^P < 0.05, ###P < 0.001 compared with the untreated cells (control group); *P < 0.05, **P < 0.01 compared with the cells treated with LPS alone (LPS group).

In terms of IL-6 production, Shapiro-Wilk test showed that the data were not normally distributed. Kruskal-Wallis test showed significant difference among different groups [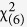 = 26.497, P < 0.001, Fig. 7]. Mann-Whitney test further showed that IL-6 production was much higher in the cells treated with LPS alone than in the control group, while okanin significantly inhibited LPS-induced IL-6 production (control group: 21.4 ± 3.3 pg/ml, LPS group: 632.4 ± 6.4 pg/ml, 1 μM group: 599.7 ± 5.1 pg/ml, 10 μM group: 529.5 ± 13.5 pg/ml, 30 μM group: 377.4 ± 3.2 pg/ml, 100 μM group: 195.8 ± 5.2 pg/ml, minocycline group: 285.9 ± 11.4 pg/ml). Regarding IL-6 mRNA, one-way ANOVA analysis revealed significant differences between the groups [F(6, 21) = 121.337, P < 0.001, Fig. 7]. Dunnett’s T3 test showed that IL-6 mRNA levels were much higher in the cells treated with LPS alone than in the control group, while okanin significantly inhibited LPS-induced IL-6 mRNA level (control group: (1.2 ± 0.1)%, LPS group: (100.0 ± 4.2)%, 1 μM group: (94.1 ± 4.7)%, 10 μM group: (67.9 ± 3.3)%, 30 μM group: (71.3 ± 3.5)%, 100 μM group: (35.6 ± 1.6)%, minocycline group: (47.3 ± 1.6)%).

In terms of TNF-α production, Shapiro-Wilk test showed that the data were not normally distributed. Kruskal-Wallis test showed significant difference among different groups [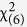 = 25.683, P < 0.001, Fig. 7]. Mann-Whitney test showed that TNF-α production was much higher in the cells treated with LPS alone than in the control group, while okanin significantly inhibited LPS-induced TNF-α production (control group: 69.0 ± 19.1 pg/ml, LPS group: 843.5 ± 4.8 pg/ml, 1 μM group: 796.3 ± 13.2 pg/ml, 10 μM group: 702.0 ± 45.2 pg/ml, 30 μM group: 491.0 ± 3.5 pg/ml, 100 μM group: 71.3 ± 3.9 pg/ml, minocycline group: 403.4 ± 3.2 pg/ml). Regarding TNF-α mRNA, Shapiro-Wilk test showed that the data were not normally distributed. Kruskal-Wallis test showed significant difference among different groups [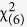 = 24.656, P < 0.001, Fig. 7]. Mann-Whitney test further revealed that LPS significantly increased mRNA expression of TNF-α, while okanin (10, 30, 100 μM) significantly inhibited LPS-induced TNF-α mRNA level (control group: (13.4 ± 0.6)%, LPS group: (100.0 ± 3.9)%, 1 μM group: (94.9 ± 5.7)%, 10 μM group: (75.3 ± 3.3)%, 30 μM group: (67.1 ± 2.6)%, 100 μM group: (26.5 ± 1.1)%, minocycline group: (75.0 ± 2.6)%).

### Effects of okanin on the activation of NF-κB signaling pathway induced by LPS

**Figure 8:**
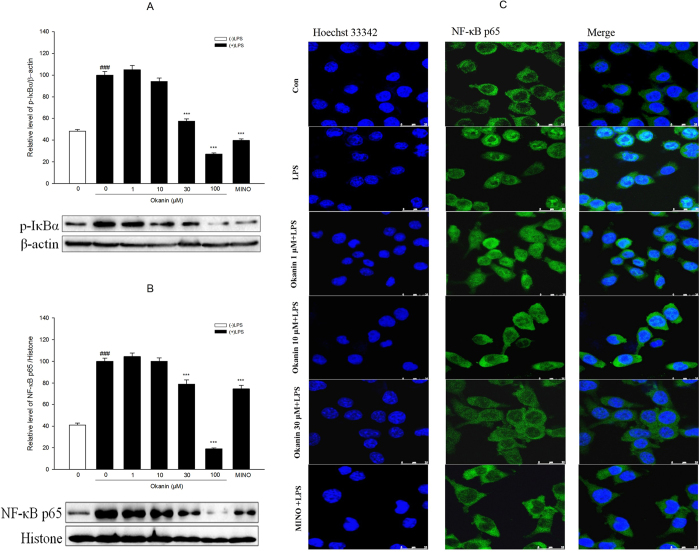
Effects of okanin on LPS-induced activation of NF-κB signaling pathway. Cells were pretreated with okanin (1, 10, 30, 100 μM) for 2 h and then stimulated with LPS for 30 min. Protein levels of p-IκBα (**A**) and NF-κB p65 (**B**) were analyzed by western blot. Full-length blots are presented in Supplementary Figures 2 and 3. The translocation of NF-κB p65 was determined by immunocytochemistry (**C**). Data are expressed as means ± SEM (n = 3). ^###^P < 0.001 compared with the control group (untreated cells); ***P < 0.001 compared with the cells treated with LPS only.

In terms of p-IκBα, one-way ANOVA analysis revealed significant differences between the groups [F(6, 14) = 155.321, P < 0.001, Fig. 8A]. The LSD test further showed that p-IκBα levels were much higher in the cells treated with LPS alone than in the control group, while okanin significantly inhibited LPS-induced p-IκBα expression (control group: (48.2 ± 1.6)%, LPS group: (100.0 ± 3.4)%, 1 μM group: (104.9 ± 3.8)%, 10 μM group: (94.2 ± 2.9)%, 30 μM group: (57.2 ± 2.2)%, 100 μM group: (27.2 ± 1.0)%, minocycline group: (39.7 ± 1.4)%).

Regarding NF-κB p65, one-way ANOVA analysis revealed significant differences between the groups [F(6, 14) = 125.447, P < 0.001, Fig. 8B]. The LSD test showed that NF-κB p65 levels were much higher in the cells treated with LPS alone than in the control group, while okanin (30, 100 μM) significantly inhibited NF-κB p65 expression in the nucleus induced by LPS (control group: (41.0 ± 1.8)%, LPS group: (100.0 ± 3.0)%, 1 μM group: (104.4 ± 3.4)%, 10 μM group: (100.1 ± 3.3)%, 30 μM group: (79.1 ± 3.7)%, 100 μM group: (19.2 ± 0.7)%, minocycline group: (74.7 ± 3.3)%), suggesting that okanin inhibited the translocation of the NF-κB p65 subunit from the cytosol to the nucleus. This result was also confirmed by immunofluorescence images, which showed that GFP-labeled NF-κB p65 was predominantly located in the cytoplasm rather than in the nucleus after okanin treatment (Fig. 8C).

### Effects of okanin on LPS-induced TLR4 expression

**Figure 9:**
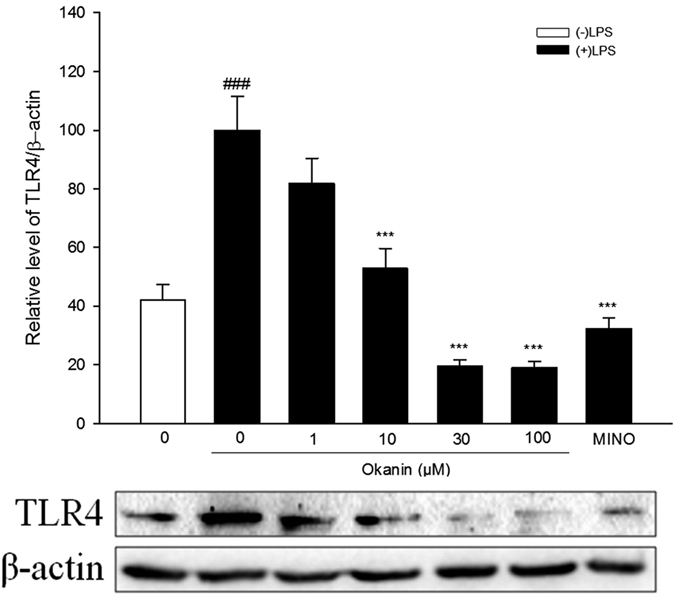
Effect of okanin on TLR4 induced by LPS in BV-2 microglial cells. Cells were pretreated with okanin (1, 10, 30, 100 μM) for 2 h and then stimulated with LPS for 24 h. Cell lysates were prepared and analyzed by western blot to detect the levels of TLR4. Full-length blots are presented in Supplementary Figure 4. Data are expressed as means ± SEM (n = 3). ^###^P < 0.001 compared with the control group (untreated cells); ***P < 0.001 compared with the cells treated with LPS only.

One-way ANOVA analysis revealed significant differences between the groups [F(6, 14) = 22.220, P < 0.001, Fig. 9]. The LSD test further showed that TLR4 levels were much higher in the cells treated with LPS alone than in the control group, while okanin (10, 30, 100 μM) significantly inhibited LPS-induced TLR4 expression (control group: (42.1 ± 5.2)%, LPS group: (100.0 ± 11.6)%, 1 μM group: (81.9 ± 8.6)%, 10 μM group: (52.7 ± 6.8)%, 30 μM group: (19.6 ± 2.0)%, 100 μM group: (19.1 ± 2.1)%, minocycline group: (32.4 ± 3.6)%).

## Discussion

Tea is a popular beverage worldwide. The different types of tea, including green tea, red tea and black tea, originate from plants of the *Camellia* genus. Recently non-*Camellia* teas, as popular health supplements, have attracted more attention from both scientists and consumers^12^. *C. tinctoria* Nutt., known as “snow chrysanthemum” or “snow tea” is traditionally used as a non-*Camellia* tea by the Uyghur folk. However, its biological activities and the mechanisms underlying its effects have not been reported in detail.

Microglia play an essential role in defending the brain against injury or disease^13^. When exposed to stimuli such as infectious agents, they become activated and begin to release pro-inflammatory molecules including NO, TNF-α, IL-1β and IL-6. Overexpression of these pro-inflammatory mediators can cause neuronal death, pathological changes and neurodegenerative diseases^14^,^15^,^16^. Inhibition of microglial activation is therefore considered to be a valid approach to prevent and treat neuroinflammation-mediated diseases^16^. Here we report that the EtOAc extract of *Coreopsis tinctoria* Nutt attenuated LPS-induced microglial activation both *in vitro* and *in vivo.* Its effective constituent, okanin, was further investigated. Okanin was able to suppress LPS-induced microglial activation. This effect may be associated with its ability to inhibit the TLR4/NF-κB signaling pathways.

To assess the anti-neuroinflammatory properties of the *C. tinctoria* extracts, we assayed NO production in BV-2 microglial cells that were stimulated with LPS. The total EtOH extract of *Coreopsis tinctoria* Nutt significantly inhibited production of NO. Further study revealed that the EtOAc extracts significantly inhibited LPS-induced NO production, while the n-BuOH extract did not. These results suggest that the EtOAc extract contained the active agent that suppresses NO production. NO is synthesized in a variety of cells and tissues by the enzyme NO synthase (NOS)^17^. Microglia express an inducible isoform of NOS (iNOS), which produces NO continuously when the cells are activated^18^. The effect of the EtOAc extract on LPS-induced iNOS mRNA expression in BV-2 microglial cells was investigated by qRT-PCR. The EtOAc extract significantly inhibited LPS-induced iNOS mRNA expression. The results may explain why the extract has the capacity to reduce NO production.

COX-2, an important enzyme associated with inflammation, has been reported to be elevated in AD brains^19^,^20^. It was previously reported that COX-2 inhibitors have a neuroprotective effect by blocking microglial activation and downstream events^11^,^21^,^22^. Here, we investigated the effect of the EtOAc extract on LPS-induced COX-2 expression in BV-2 cells. The EtOAc extract significantly inhibited LPS-induced COX-2 mRNA expression.

Following the onset of inflammation, activated microglia produce three main pro-inflammatory cytokines, namely IL-1β, IL-6 and TNF-α^23^. We used qRT-PCR to examine the effect of the EtOAc extract on LPS-induced mRNA expression of these three genes in BV-2 microglial cells. The EtOAc extract significantly inhibited LPS-induced mRNA expression of IL-1β, IL-6 and TNF-α.

We next determined the anti-neuroinflammatory effect of the EtOAc extract *in vivo*. Mice were pre-treated orally with the extract, then microglial activation was induced by intracerebroventricular administration of LPS. The microglial response was assessed immunohistochemically using an antibody against Iba-1, a protein that is strongly up-regulated in activated microglia. The EtOAc extract significantly inhibited LPS-induced microglial activation *in vivo* evidenced by the fewer Iba-1 positive cells in mice after the treatment of the EtOAc extract.

Since the EtOAc extract exhibited significant anti-neuroinflammatory effects both *in vitro* and *in vivo,* it was considered to be responsible for the potential of the traditional flower tea on overactivation of microglia. In our previous research, okanin, as the major component of EtOAc extract shown in Fig. 5, exhibited significant inhibition effect in LPS-induced microglial cells. Our previous data showed that okanin was able to block the LPS-induced production of NO by BV-2 microglia. The present study revealed that okanin was also able to inhibit the LPS-induced increase in iNOS expression. The mRNA and protein levels of iNOS were both reduced by okanin, and the level of inhibition was directly related to the concentration of okanin. Furthermore, we found that okanin lowered the mRNA and protein levels of the LPS-induced pro-inflammatory cytokines IL-6 and TNF-α. Taken together, our data showed that okanin potently inhibits a number of pro-inflammatory responses in microglial cells.

The NF-κB complex is a well-known transcriptional activator which modulates gene expression in response to a wide variety of stressors, toxins and microbial antigens, including LPS. The NF-κB complex is a homodimer or heterodimer of proteins in the NF-κB and Rel families^24^. Under normal conditions, NF-κB associates with members of the cytoplasmic IκB family of inhibitor proteins. Exposure of the cell to stimuli such as LPS triggers receptor-mediated phosphorylation of IκB, which then undergoes ubiquitination followed by proteasomal degradation^25^. Meanwhile, the NF-κB dimer shuttles into the nucleus, where it binds to specific response elements upstream of its target genes, thus enhancing transcription^24^. As mentioned above, the NF-κB complex is a key mediator of the LPS-induced activation of microglia, and activates the transcription of genes encoding cytokines^11^,^26^. Therefore, we used western blotting and immunofluorescence assays to test whether okanin affects the NF-κB subunit p65 (RelA) and the NF-κB inhibitor IκB in BV-2 cells treated with LPS. The data showed that okanin significantly inhibited the LPS-induced phosphorylation of IκBα in BV-2 cells. Furthermore, in LPS-treated BV-2 cells, okanin significantly suppressed the shuttling of p65 into the nucleus.

Toll-like receptors (TLRs) recognize molecules, such as cell membrane products and nucleic acids, which are found on different kinds of microbial pathogen^27^,^28^. Most TLR-initiated signaling causes NF-κB to shuttle into the nucleus, as described above, where it activates a variety of genes that encode pro-inflammatory factors^24^. In the CNS, TLRs 1–9 are expressed on microglia^29^. TLR4 is an innate immune receptor, which recognizes LPS in the outer membrane of gram-negative bacteria^30^,^31^. It has been shown that preventing TLR4-mediated signaling suppresses the activation of microglia and reduces the release of pro-inflammatory cytokines^31^. Our work demonstrates that the LPS-induced activation of BV-2 microglial cells was associated with up-regulated expression of TLR4, which could be decreased by okanin. This suggests that down-regulated expression of TLR4 might contribute to the inhibitory effect of okanin on the activation of BV-2 microglia.

In conclusion, this study shows for the first time that the EtOAc extract of *Coreopsis tinctoria* Nutt attenuates LPS-induced microglial activation both *in vitro* and *in vivo.* Its effective constituent, okanin, suppresses LPS-induced microglial activation. This effect may be associated with its ability to inhibit the TLR4/NF-κB signaling pathways. Considering that neuroinflammation mediated by activated micoglia plays an important role in neurodegenerative diseases, okanin is a potential treatment for neurodegenerative diseases.

## Materials and Methods

### General

UPLC-PDA analysis was performed on a Waters Acquity UPLC H-Class system fitted with an Acquity H-class BEH-C18 column (1.7 μm, 100 × 2.1 mm) (Waters Corporation, Milford, MA, USA). GSH was produced by Bailingwei Group Co. of China. Lipopolysaccharide (LPS, from E. coli serotype 055:B5) was obtained from Sigma Chemical Co. (St. Louis, MO, USA). Fetal bovine serum (FBS) was obtained from Tianjinhaoyang Biological Manufactory Co., Ltd (Tianjin, China). Dulbecco’s Modified Eagle’s Medium (DMEM) was obtained from Gibco BRL (Grand Island, NY, USA). (3-[4,5- Dimethylthiazol-2-yl]-2,5-diphenyltetrazolium bromide (MTT) was obtained from Beyotime Biotechnology (Beijing, China). Trizol was obtained from Invitrogen Co. (Carlsbad, CA, USA). Go Taq^®^qPCR Master Mix was purchased from Promega Corporation (Madison, WI, USA). Antibodies specific for TLR4, NF-κB p65 and phospho-IκBα were from Cell Signaling Technology, Inc. (Danvers, MA, USA). Anti-Iba1 antibody was purchased from Millipore (Temecula, CA, USA).

### Plant material

Flowers of *Coreopsis tinctoria* Nutt. were collected in Kunlun Mountain in Xinjiang and identified by Professor Xiaoguang Jia, XinJiang Institute of Chinese Materia Medica. Voucher specimen (NO. 20130912) is deposited in the School of Traditional Chinese Material Medica, Shenyang Pharmaceutical University.

### Preparation of Okanin

Okanin (30 mg) was prepared from the EtOAc extract of flower tea *C. tinctoria* Nutt. according to the method described in our previous work^5^. Its structure was identified on the basis of NMR spectra analysis.

### Determination of the okanin content in EtOH, EtOAc and n-BuOH extracts of of *C. tinctoria* Nutt

Appropriate amounts of okanin were dissolved in methanol. The EtOH extract, EtOAC extract (100 mg) and *n-*BuOH extract (100 mg) were extracted with 5 ml MeOH for 5 minutes by sonication. After filtration through a 0.22 μm membrane, an aliquot of 1 μL of each solution was analyzed using a Waters Acquity UPLC H-Class system equipped with a Sample Manager-FTN, Quaternary Solvent Manager, PDA Detector and Empower software. Acquity H-class BEH-C18 column (1.7 μm, 100 × 2.1 mm) was employed at a flow rate of 0.4 mL/min. The mobile phases were water containing 0.1% formic acid (solvent A) and acetonitrile (solvent B) delivered in a 4.5 min gradient consisting of the following: 0–2.3 min, 12–20%B, 2.3–4.5 min, 20–72% B. The column compartment was kept at a temperature of 35 °C. The detector wavelength range was set from 200 nm to 400 nm.

### Cell culture

The murine microglial cell line BV-2 was used in the present study. BV-2 cells were cultured in DMEM supplemented with 10% FBS, 2 mM glutamine, 100 U/ml penicillin, and 100 μg/ml streptomycin at 37 °C in humidified 5% CO_2_.

### Measurement of cell viability

Cell viability was measured with MTT assays^6^,^32^. BV-2 cells were seeded into 96-well plates, incubated with extracts of *C. tinctoria* Nutt or okanin in the presence of LPS (100 ng/mL) for 24 h, then treated with 0.25 mg/ml MTT at 37 °C for 4 h. After the supernatant was removed, the formazan crystals produced by reduction of MTT were dissolved by adding DMSO. The proportion of viable cells was determined by colorimetric assay in a plate reader (Bio-Tek, Winooski, VT, USA) at 490 nm.

### Nitrite assay

The level of nitrite (NO_2_^−^) in the supernatant of cultured cells was determined using the Griess reaction^6^,^32^. BV-2 cells in 96-well plates were treated with extracts of *C. tinctoria* Nutt in the presence of LPS (100 ng/mL) for 24 h, then 50 μl supernatant was added to 50 μl Griess reagent. The solutions were mixed and incubated for 15 min at room temperature. The absorbance of the samples was then determined with a plate reader (Bio-Tek, Winooski, VT, USA) at 540 nm.

### Quantitative real-time PCR (qRT–PCR)

For detection of mRNA expression of pro-inflammatory factors by qRT-PCR, BV-2 microglial cells were pretreated with extract of *C. tinctoria* Nutt or okanin for 2 h, then exposed to LPS for 4 h. Total RNA was extracted from the cells with Trizol reagent. After cDNA synthesis, qRT-PCR assays were carried out using a GoTaq one-step real-time PCR kit with SYBR green on a CFX Connect^TM^ real-time PCR system (Bio-Rad, Hercules, CA, USA). Expression levels of iNOS, COX-2, IL-1β, IL-6 and TNF-α were normalized to those of GAPDH (see Table 1 for primer sequences).

### Enzyme-linked immunosorbent assay (ELISA)

The protein levels of IL-6 and TNF-α were determined using ELISA. BV-2 cells were pretreated with okanin for 2 h and then stimulated with 100 ng/ml LPS for 24 h. The levels of IL-6 and TNF-α in the culture medium were measured by ELISA kits as previously reported^33^. Briefly, standards or samples were added to the wells of the microplate pre-coated with a specific monoclonal antibody. After washing away any unbound substances, an enzyme-linked polyclonal antibody was pipetted into the wells. The substrate solution was added to the wells after a wash to remove any unbound antibody-enzyme reagent. The color development was finally stopped and the absorbance was read at 450 nm using a plate reader (Bio-Tek, Winooski, VT, USA).

### Western blot analysis

Western blotting was carried out as previously reported^11^,^34^,^35^. To detect NF-κB p65 and phosphorylation of IκBα, BV-2 cells were pretreated with okanin for 2 h and then exposed to LPS for 30 min. To detect TLR4, BV-2 cells were pretreated with okanin for 2 h and then exposed to LPS for 24 h. Cells were lysed in lysis buffer after washing in ice-cold PBS^35^. The protein content of the lysates was measured using the BCA assay, then the samples were separated by 12% SDS-PAGE. The proteins were transferred to a membrane, which was incubated sequentially with blocking buffer, primary antibodies, and horseradish peroxidase-conjugated secondary antibodies.

### Immunofluorescence staining

The nuclear localization of NF-kB p65 was examined by immunofluorescence assay using a confocal laser scanning microscope (Leica, Wetzlar, HE, Germany). BV-2 cells were pretreated with okanin for 2 h. After stimulation with LPS for 30 min, the cells were fixed, permeabilized and blocked. The polyclonal antibody to NF-kB p65 was applied overnight, followed by 1 h of incubation with fluorescein isothiocyanate (FITC)-conjugated anti-rabbit IgG. After being washed with PBS, the cells were visualized and photographed.

### Molecular docking

The molecular docking study was carried out according to the method described in our previous work^33^. Briefly, it was performed using AutoDock4.2. The structure of iNOS in complex with an imidazopyridine inhibitor (PDB code 3NW2) was applied in the simulation. The co-crystal ligand was used to define the binding site. The docking grid was assigned around the active site. Nine docking calculations were carried out. The generated poses were clustered and the top-ranked compounds were visually inspected.

### Animals

All protocols involving animals were performed according to the Regulation of Experimental Animal Administration from the State Committee of Science and Technology of the People’s Republic of China. Male Swiss-Kunming mice were provieded by the Experimental Animal Center of Shenyang Pharmaceutical University. The body weight of the animals was 18–22 g. The mice were housed under standard conditions in an animal facility with a light:dark cycle of 12:12 h. Access to food and water was unrestricted. At least five days before the experiments started, the animals were allowed to become familiar with the laboratory environment. The experiment was performed under the approval of the Committee of Experimental Animal Administration of Shenyang Pharmaceutical University (permission number # SYPU-IACUC-C2015-0427-101).

### Treatment of mice with the EtOAc extract of *C. tinctoria* Nutt

Mice were randomly allocated to three treatment groups. Mice in the control group were treated orally with vehicle and injected with PBS. Mice in the LPS groups were treated orally with vehicle and injected with LPS. Mice in the EtOAc group were treated orally with the EtOAC extract of *C. tinctoria* Nutt. and injected with LPS. All groups received daily oral administration of vehicle or extract of *C. tinctoria* Nutt at a dose of 200 mg/kg/day. The animals were injected intracerebroventricularly with LPS (40 μg/mouse) or PBS on day 7 and were sacrificed 3 days later. Seven mice were used in each group.

### Immunohistochemical analysis of mouse brain sections

To prepare brain tissues for immunohistochemistry, mice were first anesthetized with chloral hydrate (400 mg/kg, intraperitoneal injection). The animals were then perfused transcardially with heparin (10 U/ml in saline solution) followed by paraformaldehyde (4% in 0.1 M phosphate buffer, pH 7.4). The brains were removed and incubated in paraformaldehyde for 8 h, then in 30% sucrose solution for 24 h. The samples were stored at −20 °C until required.

Coronal sections (20 μm) from the hippocampal region were cut on a freezing microtome (LEICA CM1850), then washed in 10 mM PBS (pH 7.4). Slides and sections were then treated as reported previously^36^,^37^. Cells positive for Iba-1 staining were counted under a light microscope (LEICA DMI 3000 B). The observation of immunohistochemistry was obtained from two sections for each animal. The researcher was blinded to the treatment status of the slides.

### Statistical analysis

Statistical analyses were carried out with SPSS 17.0 software (SPSS Inc., Chicago, IL, USA). Data distribution was tested by Shapiro-Wilk test. In the case of normal distribution, data were analyzed by ANOVA followed by Dunnett’s T3 test or Fisher’s least significant difference (LSD) test. In other case, Kruskal-Wallis test and Mann–Whitney test were performed. Two sided tests were applied. Results are presented as mean ± SEM. Differences were considered statistically significant at P < 0.05.

## Additional Information

**How to cite this article:** Hou, Y. *et al*. Okanin, effective constituent of the flower tea *Coreopsis tinctoria*, attenuates LPS-induced microglial activation through inhibition of the TLR4/NF-κB signaling pathways. *Sci. Rep.* **7**, 45705; doi: 10.1038/srep45705 (2017).

**Publisher's note:** Springer Nature remains neutral with regard to jurisdictional claims in published maps and institutional affiliations.

## Supplementary Material

Supplementary Information

## Figures and Tables

**Table 1:** Primer sequence used in qRT-PCR assay.

Gene	Forward primer	Reverse primer
iNOS	5′-GGCAAACCCAAGGTCTACGTT-3′	5′-GAGCACGCTGAGTACCTCATTG-3′
COX-2	5′-AAGACGCCACATCCCCTATT-3′	5′-ACAGAATGCGTAGAGAGGGG-3′
IL-1β	5′-TGACGGACCCCAAAAGATGA-3′	5′-TCTCCACAGCCACAATGAGT-3′
IL-6	5′-TAGTCCTTCCTACCCCAATTTCC-3′	5′-TTGGTCCTTAGCCACTCCTTC-3′
TNF-α	5′-CCCTCACACTCAGATCATCTTCT-3′	5′-GCTACGACGTGGGCTACAG-3′
GAPDH	5′-AGGTCGGTGTGAACGGATTTG-3′	5′-TGTAGACCATGTAGTTGAGGTCA-3′
