# Supplementary material for: Okanin, effective constituent of the flower tea Coreopsis tinctoria, attenuates LPS-induced microglial activation through inhibition of the TLR4/NF-κB signaling pathways
Source: Sci Rep. 2017 Apr 3;7:45705. doi: 10.1038/srep45705 (PMC5377376; doi:10.1038/srep45705)

Okanin, effective constituent of the flower tea *Coreopsis tinctoria*, attenuates LPS-induced microglial activation through inhibition of the TLR4/NF-κB signaling pathways

Yue Hou[1], Guoxun Li[1], Jian Wang[2], Yingni Pan[3], Kun Jiao[1], Juan Du[1], Ru Chen[1], Bing Wang[1,]*& Ning Li[3,]*

[1]College of Life and Health Sciences, Northeastern University, Shenyang 110819, China

[2]School of Pharmaceutical Engineering, Shenyang Pharmaceutical University, Shenyang 110016, China

[3]School of Traditional Chinese Materia Medica, Key Laboratory of Structure-Based Drug Design and Discovery, Shenyang Pharmaceutical University, Ministry of Education, Shenyang 110016, China

Supplementary Figure 1A. Full-length blots of iNOS (up) and β-actin (down).

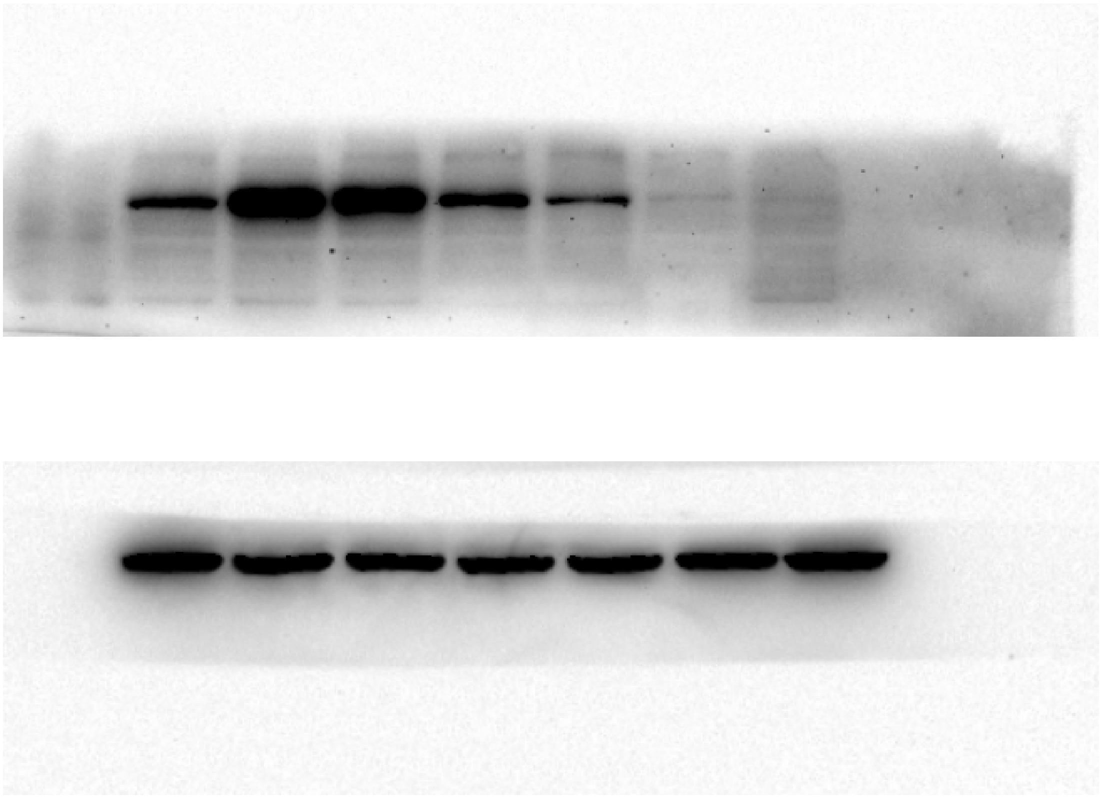

LPS	–	+	+	+	+	+	+
Okanin (μM)	–	–	1	10	30	100	–
MINO (μM)	–	–	–	–	–	–	30

Supplementary Figure 1B. Multiple exposures of full-length blots of iNOS.

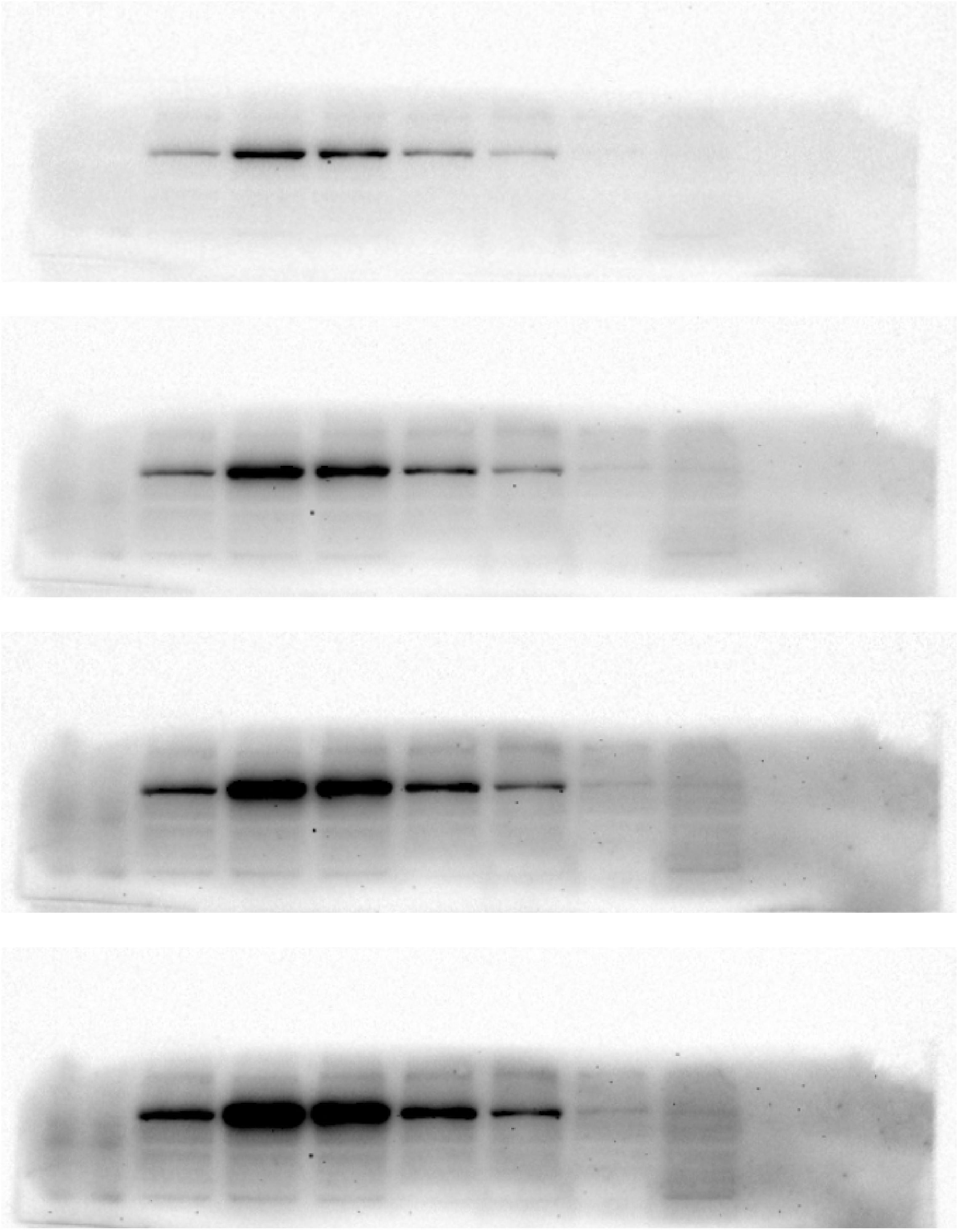

LPS	–	+	+	+	+	+	+
Okanin (μM)	–	–	1	10	30	100	–
MINO (μM)	–	–	–	–	–	–	30

Supplementary Figure 2A. Full-length blots of p-IκBα (up) and β-actin (down).

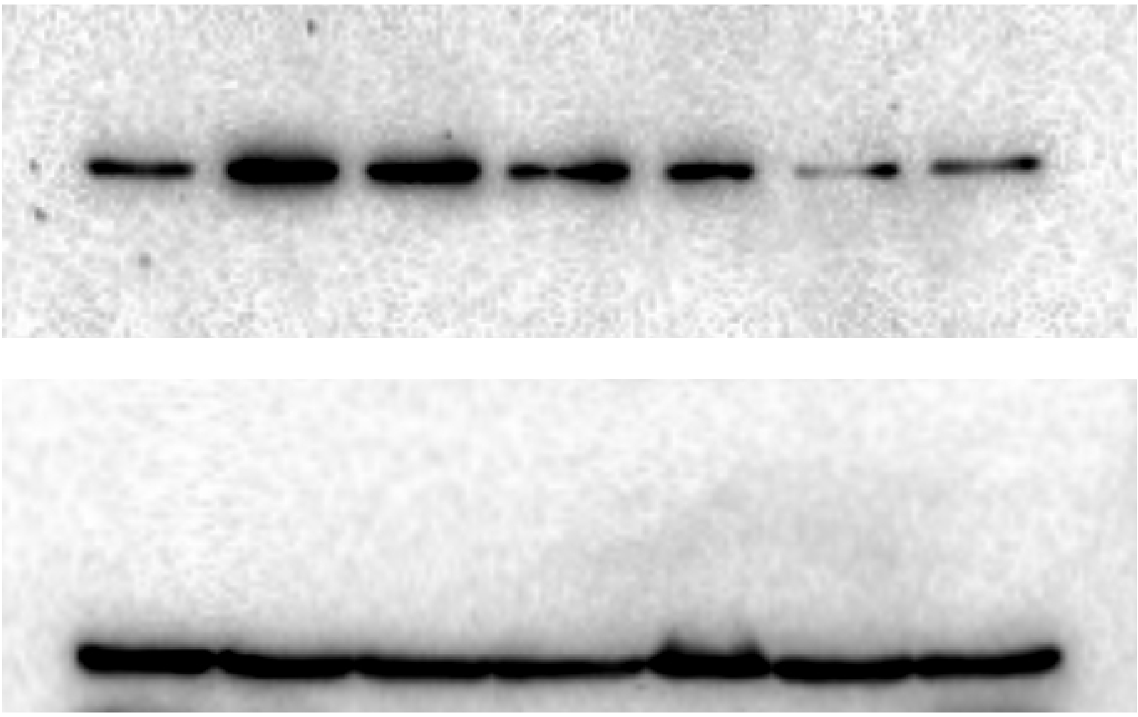

LPS	−	+	+	+	+	+	+
Okanin (μM)	−	−	1	10	30	100	−
MINO (μM)	−	−	−	−	−	−	30

Supplementary Figure 2B. Multiple exposures of full-length blots of p-IκBα.

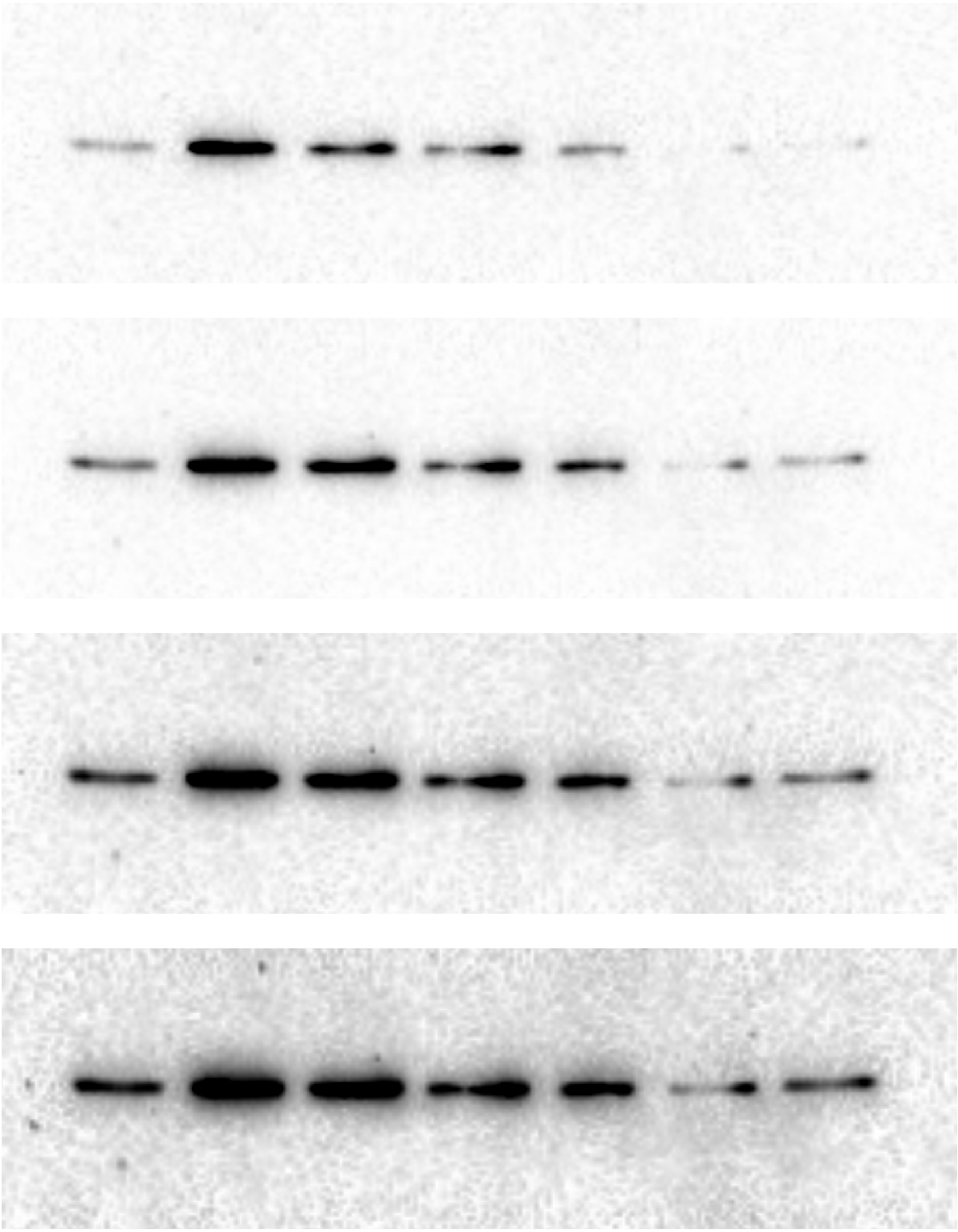

LPS	−	+	+	+	+	+	+
Okanin (μM)	−	−	1	10	30	100	−
MINO (μM)	−	−	−	−	−	−	30

Supplementary Figure 3A. Full-length blots of NF-κB p65 (up) and Histone (down).

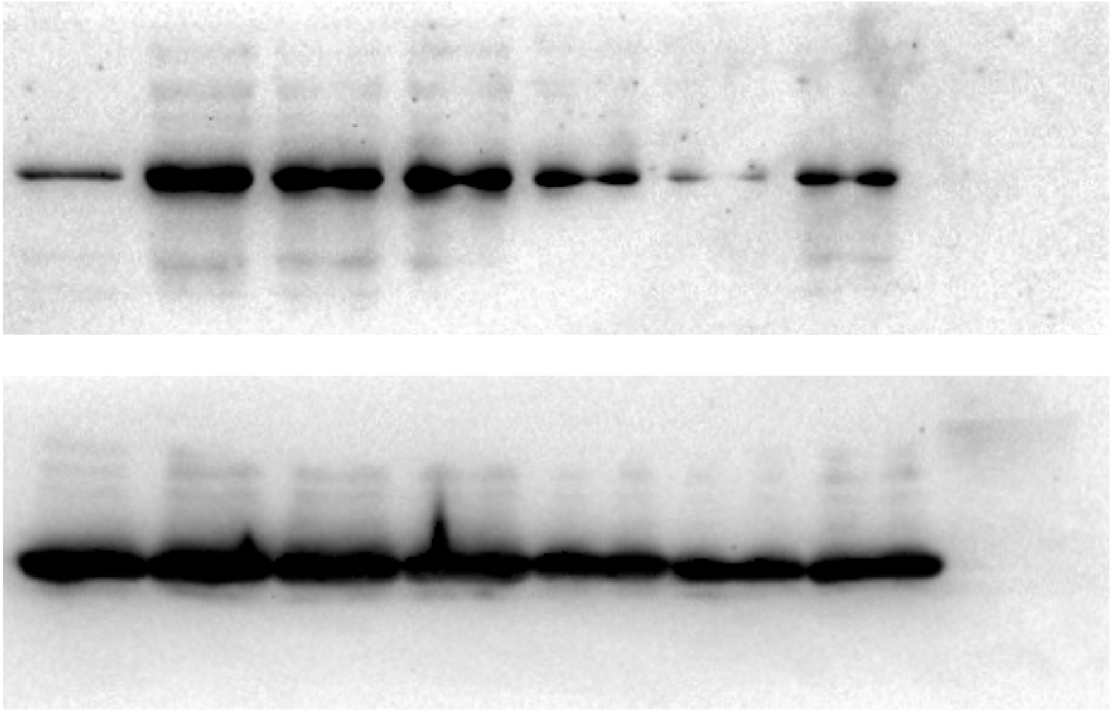

LPS	−	+	+	+	+	+	+
Okanin (μM)	−	−	1	10	30	100	−
MINO (μM)	−	−	−	−	−	−	30

Supplementary Figure 3B. Multiple exposures of full-length blots of NF-κB p65.

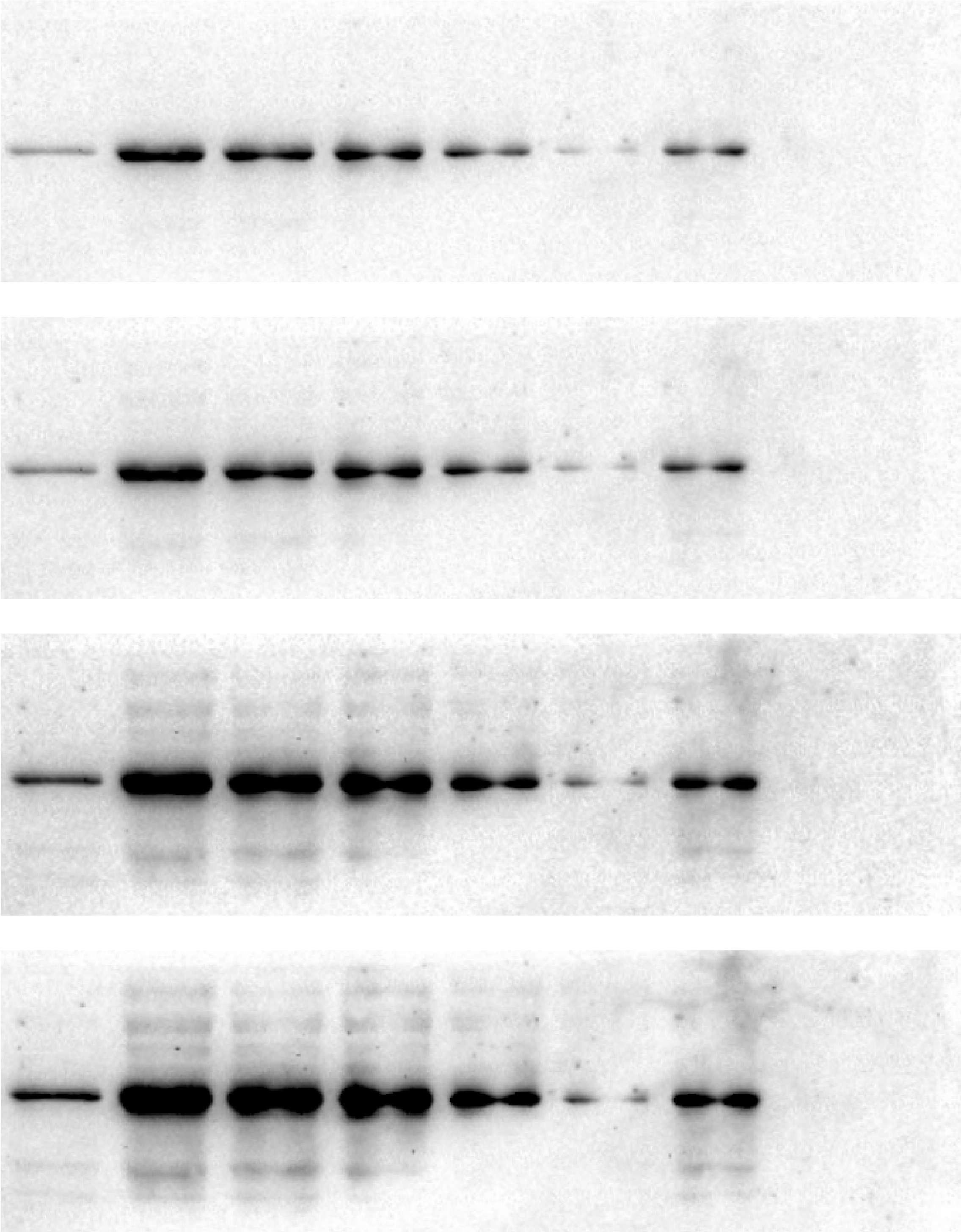

LPS	–	+	+	+	+	+	+
Okanin (μM)	–	–	1	10	30	100	–
MINO (μM)	–	–	–	–	–	–	30

Supplementary Figure 4A. Full-length blots of TLR4 (up) and β-actin (down).

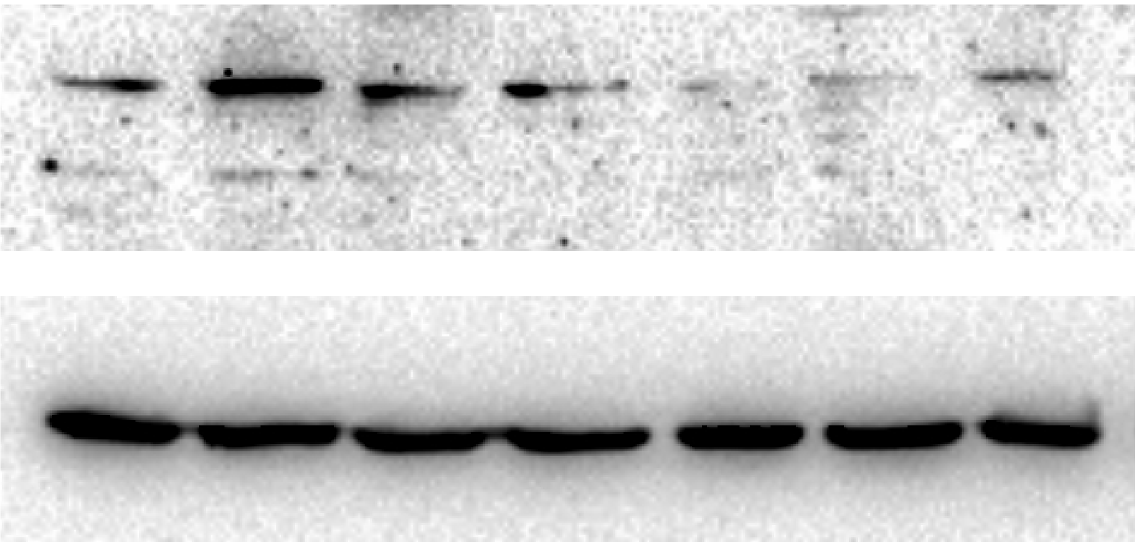

LPS	—	+	+	+	+	+	+
Okanin (μM)	—	—	1	10	30	100	—
MINO (μM)	—	—	—	—	—	—	30

Supplementary Figure 8. Multiple exposures of full-length blots of TLR4.

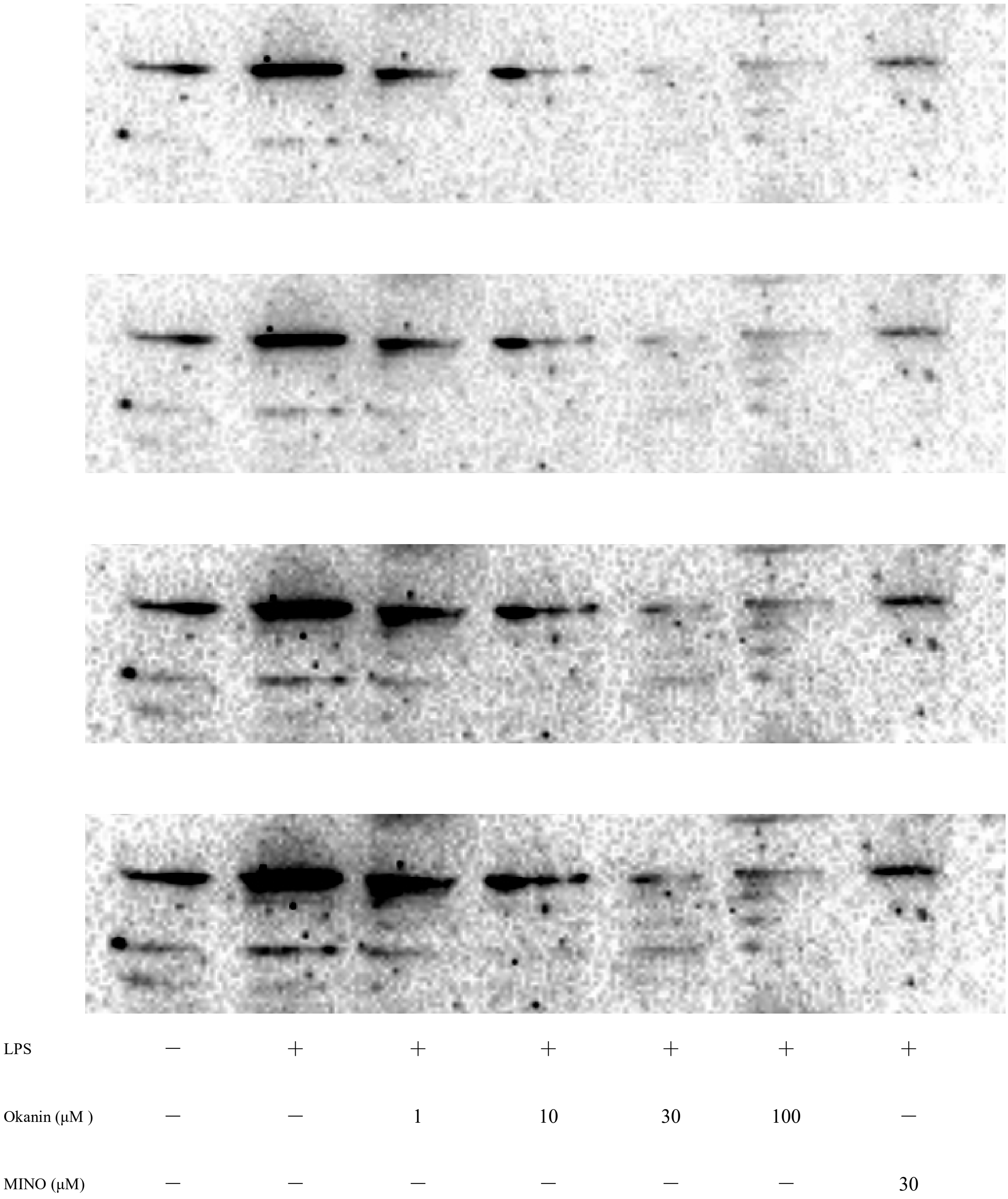

Supplement: Supplementary Information [file srep45705-s1.pdf]
